# Two-Photon Sub-Bandgap Photocurrent in Surface-Nanotextured Black Diamond Films for Solar Energy Conversion

**DOI:** 10.1021/acsphotonics.5c00722

**Published:** 2025-08-11

**Authors:** M. Girolami, A. Bellucci, M. Mastellone, S. Orlando, S. Pettinato, V. Serpente, S. Salvatori, D. M. Trucchi

**Affiliations:** a DiaTHEMA Lab, Istituto di Struttura della Materia, Consiglio Nazionale delle Ricerche (ISM - CNR), Sede Secondaria di Montelibretti, Strada Provinciale 35D 9, Montelibretti, Roma 00010, Italy; b Istituto di Struttura della Materia, Consiglio Nazionale delle Ricerche (ISM - CNR), Sede Secondaria di Tito Scalo, Area Industriale - Contrada S. Loia, Tito Scalo, Potenza 85050, Italy; c Faculty of Engineering, Università degli Studi Niccolò Cusano, Via don Carlo Gnocchi 3, Roma 00166, Italy

**Keywords:** defect-engineering, diamond, surface nanotexturing, two-photon sub-bandgap photocurrent, solar energy conversion

## Abstract

Two-photon sub-bandgap photocurrent (TPPC) is demonstrated in surface-nanotextured black diamond films fabricated by a two-step femtosecond laser treatment at an optimal total accumulated laser fluence of 5.0 kJ cm^–2^, unequally split between the two steps with a split ratio of 2:1. A broad intermediate band of electrically active deep-level defects, located at an energy distance of 1.83 eV from the conduction band, is experimentally observed by sub-bandgap spectral response evaluation in the 190–1000 nm wavelength range, allowing for a significant enhancement of quantum efficiency under photovoltaic conditions. This work represents the first experimental demonstration of TPPC in bulk materials with deep-level impurities conceived for intermediate-band solar cells and provides a solid physical interpretation of the operating principle of defect-engineered black diamond-based devices for solar energy conversion.

## Graphical abstract

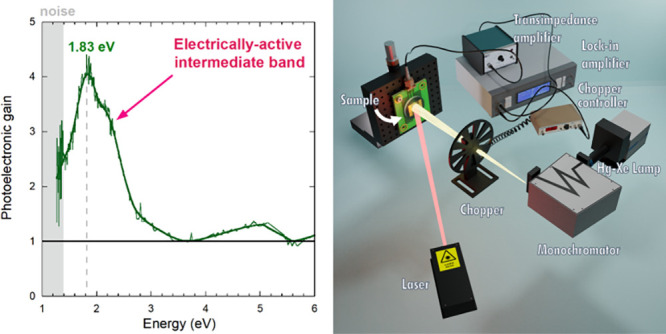

## Introduction

In recent years, defect engineering, which is a process of the controlled creation of defects in the bulk volume and/or on the surface of a material (including nanomaterials), has been demonstrated to be an extremely powerful strategy to tailor the physical and chemical properties to a specific application. In addition to the obvious structural and morphological modifications, the introduction of defects indeed allows for the design of a new electronic structure, implying significantly different optical, electrical, electrochemical, and optoelectronic properties.

In this context, the extremely versatile nature of carbon, due to its unique properties such as tetra-valency and catenation, implying the ability to form a huge variety of stable compounds and a high number of possible bond combinations at different places, makes carbon-based materials perfect candidates for the development of defect-engineered devices for energy-related applications.^1–3^ One remarkable example is given by the “black diamond” technology for solar energy conversion^4^ based on the defect engineering of diamond substrates by femtosecond laser treatment. Ultrafast laser pulses with an energy density higher than the diamond ablation threshold are indeed able to induce periodic 1D or 2D surface nanostructures, which are demonstrated to drastically modify both the optical and the photoelectronic properties of the pristine material. First, native semitransparent diamond, which is a wide-bandgap (about 5.5 eV) semiconductor, turns black, increasing its solar absorptance up to 99% in the 200–2000 nm wavelength range;^5^ in addition, a significant increase of quantum efficiency has been observed^4,6^ in the UV–vis–NIR range (250–900 nm), implying the ability of converting the absorbed photons into charge carriers exploitable for electrical power generation. This remarkable enhancement of the interaction of black diamond with solar photons is strictly connected with the laser-induced introduction of defect-related energy levels within the bandgap of the pristine diamond, forming a so-called “intermediate-band” (IB).

The concept of IB can be exploited for the fabrication of solar cells (IBSCs), potentially able to exceed the efficiency limit (Shockley–Queisser limit) of conventional single-bandgap solar cells.^7,8^ The main physical phenomenon underlying the operating principle of IBSCs is the production of two-photon sub-bandgap photocurrent (TPPC), i.e., obtained through the absorption of two photons with an energy lower than the semiconductor bandgap. Several attempts of IBSC prototypes have been already reported exploiting different technologies, namely, quantum dots^9^ (QDs), highly mismatched alloys^10^ (HMAs), organic molecules^11^ (OMs), and bulk materials with deep-level impurities^12^ (DLIs); however, TPPC has been demonstrated so far only for QD- and HMA-based devices,^13–16^ whereas no experimental evidence of TPPC has been reported for IBSCs based on OM and bulk materials with DLI. However, while defect engineering of OM-based devices is still in its infancy, bulk semiconductors with DLI have been thoroughly investigated in the literature on IBSCs, coming up to the conclusion that a very large density of DLI is necessary to induce an effective intermediate band,^17^ with the consequence in most cases of unacceptably degrading both the structural quality and the electronic properties of the host material. Unfortunately, this makes the experimental investigation of TPPC very challenging: indeed, even if nonradiative recombination can be suppressed at high defect concentrations,^18,19^ promoting the photogeneration of electron–hole pairs instead of heat, the sensible degradation of charge transport properties actually quenches photocurrent, thus nullifying the effectiveness of the intermediate band. In other words, the intermediate band, not contributing to an effective increase of the photogenerated current, is only optically and electronically active but not electrically.

Black diamond belongs to the family of bulk materials with DLI but represents a significant exception: when the density of DLI induced by laser surface nanotexturing is within an optimal range, corresponding to an optimal value of accumulated laser fluence, the charge transport properties of the host material do not degrade but can even be enhanced; for instance, the mobility-lifetime product of photogenerated carriers, which is one of the most important figures of merit of a material conceived for photoconductive devices, is higher in black than in semitransparent diamond,^4,6^ leading to the formation of an effective “electrically active” intermediate band, hence paving the way to a possible demonstration of TPPC. In this work, taking advantage of the progress made in recent years on black diamond fs-laser-based technology, we provide the first experimental evidence of two-photon sub-bandgap photocurrent response in a bulk material with deep-level defects, closing the gap on quantum dots and highly mismatched alloys in the path toward intermediate-band photovoltaic solar absorbers.

## Experimental Section

Two freestanding CVD polycrystalline 500 μm thick circular (10 mm diameter) diamond samples, provided by Element Six Ltd.,^20^ were used for this work. The first one was subjected to a surface two-step laser treatment according to the procedure described elsewhere,^5^ which is briefly recalled in the following, whereas the second one was left untreated and used as a reference sample. The treatment was performed with a femtosecond pulsed laser beam (800 nm wavelength, 3.6 mJ pulse energy, 100 fs pulse duration, 1 kHz repetition rate), delivering to the sample a total accumulated laser fluence of 5.0 kJ cm^-2^, demonstrated to be effective in obtaining black diamond films with the best performance in terms of integrated solar quantum efficiency.^4^ The laser spot (250 μm diameter) scanned the sample surface in a two-step process: in the first step, the surface was raster scanned along the horizontal (*x*) axis, whereas in the second step, the raster scan was performed along the vertical (*y*) axis. Aimed at obtaining a “2D-like” pseudoperiodic nanotexturing,^5^ which allows for the maximization of solar absorptance (>98% in the 200–2000 nm wavelength range), the accumulated laser fluence was unequally split between the two scanning steps in order to balance the incubation effect (i.e., the instantaneous increase of the absorption coefficient caused by laser pulses during the first step). The split ratio was set to 2:1 (i.e., 66.6% of the accumulated fluence delivered in the first step and 33.3% in the second step).

Before each laser treatment, the sample was subjected to the following cleaning procedure in order to remove possible nondiamond contents, residual debris, and organic and metallic contaminants: (i) wet etching in a strongly oxidizing solution (H_2_SO_4_:HClO_4_:HNO_3_ in a 1:1:1 ratio, 30 min at boiling point); (ii) ultrasound sonication in acetone and isopropanol for 15 min; (iii) rinsing with deionized water; (iv) drying in pure nitrogen flow. The same cleaning procedure was adopted for the untreated sample.

For the evaluation of the photoelectronic properties, the same metal–semiconductor–metal (MSM) structure was employed for both samples (Figure 1a). In this way, the device works as a photodetector with a “sandwich” configuration (i.e., with one electrode on the top surface and the other one on the bottom surface), which should be preferred to “coplanar” geometry (i.e., with both electrodes on the top surface) to exploit the lower dark current of the underlying bulk diamond with respect to its surface.^21^ Specifically, in a “sandwich” configuration, photogenerated carriers are separated by a uniform electric field acting on the whole thickness of the diamond sample and collected by the metal contacts, thus producing photocurrent (Figure 1b). Metal electrodes (200 nm-thick Al layers covered by a 50 nm-thick Au layer to prevent Al oxidation) were fabricated on the top and bottom surfaces of each sample by RF magnetron sputtering followed by a standard photolithographic process. Specifically, the bottom electrode is a 5 × 5 mm^2^ square, whereas the top electrode consists of two short-circuited interdigitated electrodes (100 μm width, 100 μm spacing), resulting in an overall active area of 12.5 mm^2^. The interdigitated structure has a 2-fold purpose: (i) allow the shorter wavelength photons to be effectively absorbed by the diamond bulk and not by the top metal electrodes; (ii) form an asymmetric contact geometry between the top and the bottom electrode to ensure operation with no need for external bias, as it will be described in the next section. The samples were finally mounted on a standard PCB board equipped with an SMA connector. The bottom electrode was grounded, whereas the top electrode was used for signal collection.

**Figure 1:**
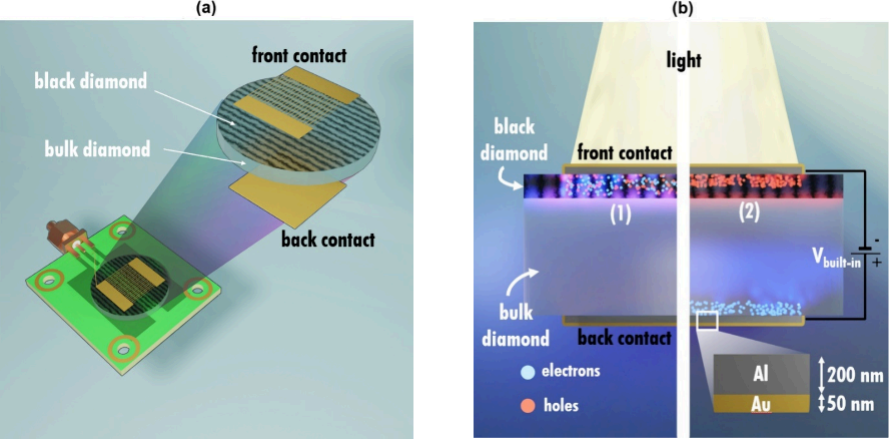
(a) Sketch of the MSM structure employed for the photocurrent measurements on the black diamond sample (in the case of the pristine sample, the front contact lies directly on bulk diamond). (b) Operating principle of the device with a “sandwich” configuration: charge carriers are photogenerated (1) in the black diamond film (or directly in bulk diamond in the case of the pristine sample), and then electrons and holes are separated (2) by the built-in electric field and collected by the respective metal contacts, generating photocurrent.

## Results and Discussion

### One-Photon Spectral Photocurrent Measurements

The photoelectronic response of the two diamond samples (the black and the pristine one) was preliminary evaluated in the 190–1000 nm wavelength range by means of standard one-photon modulated photocurrent measurements. The experimental setup is shown in Figure 2.

**Figure 2:**
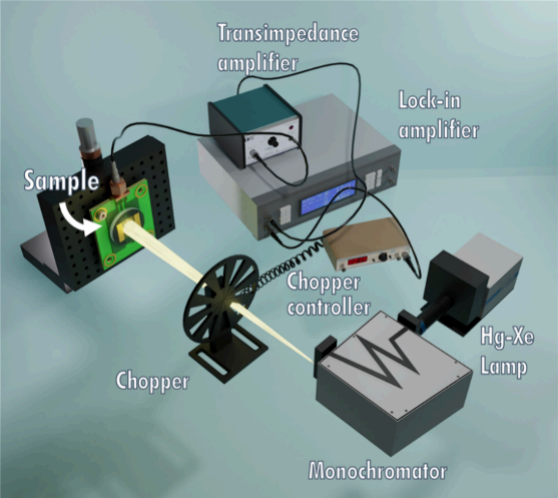
Sketch of the experimental setup for the one-photon spectral photocurrent measurements.

Light came out from the output slit of a monochromator (Newport Cornerstone 260) coupled to a Hg–Xe lamp, modulated at a frequency of 14 Hz by a SR540 mechanical chopper, and focused on the active area of the device. A transimpedance amplifier (Princeton Applied Research 181) converted the photogenerated current into a voltage measured by a lock-in amplifier (EG&G 5209). Power was measured with a calibrated Si-based photodiode (Newport 818-UV) and a power meter (Newport 843-R). Measurements were performed in self-powered mode, i.e., with no external bias applied, exploiting, for charge separation and collection, the built-in electric field induced by the back-to-back Schottky barrier configuration of the asymmetric Al electrodes. As indeed reported on diamond-based self-powered photodetectors^22^ employing the same MSM “sandwich” configuration as our device, two asymmetric Al electrodes on the top and bottom surfaces of the sample (i.e., back-to-back) form two Schottky contacts with different barrier heights, establishing a built-in electric field within the diamond bulk, the intensity of which depends on the difference between the two barrier heights. When the device is illuminated, the barrier height of the top contact decreases due to intense photogeneration beneath the light-receiving surface, hence increasing the asymmetricity with the bottom contact: as a result, the built-in electric field is more intense, which is essential for effective self-powered operation.

External quantum efficiency, defined as EQE­(λ) *=* (*hc*/*q*λ*)* × (|I_ph_(λ)|/*P*(λ)), where λ is the output wavelength of the monochromator, *h* is the Planck’s constant, *c* is the speed of light, *q* is the electronic charge, |I_ph_(λ)| is the photocurrent amplitude, and *P*(λ) is the incident power, was measured for both samples. As can be seen from Figure 3a, in both cases, a sub-bandgap (i.e., for λ > 227 nm, corresponding to the diamond energy bandgap *E*_gap_ of 5.46 eV) photocurrent is measured, even in the pristine one. However, this should not be surprising, with both samples having a polycrystalline structure, hence with a certain density of both point and extended defects (e.g., grain boundaries), inducing a broad tail of defect-related energy levels within the bandgap, which can contribute to photocurrent generation through trap-assisted charge transport. More interestingly, the EQE of the black diamond sample is higher than that of the pristine one in the 400–900 nm wavelength range. The main purpose of our research on laser-induced defect engineering of CVD diamond is indeed to find a possible technological way, starting from a given distribution of defects within the bandgap of the pristine material, to modify it properly, with the aim of increasing the density of electrically active defects (i.e., not only able to absorb photons but also to efficiently contribute to photocurrent generation) in the wavelength range of high sunlight irradiance (around 550 nm). It should be however observed that, even if the optical absorptance of the black diamond exceeds 98% in this range^5^ (whereas that of the pristine one is only 20%), the enhancement of EQE is only slight. That is because, while the introduction of defect states is always beneficial to optical absorption, in order to obtain a similar enhancement of EQE, the defect states should necessarily act as fast traps with short re-emission times, and not as recombination centers, which capture photocarriers for a long time and do not result useful to photocurrent generation.^4^

**Figure 3:**
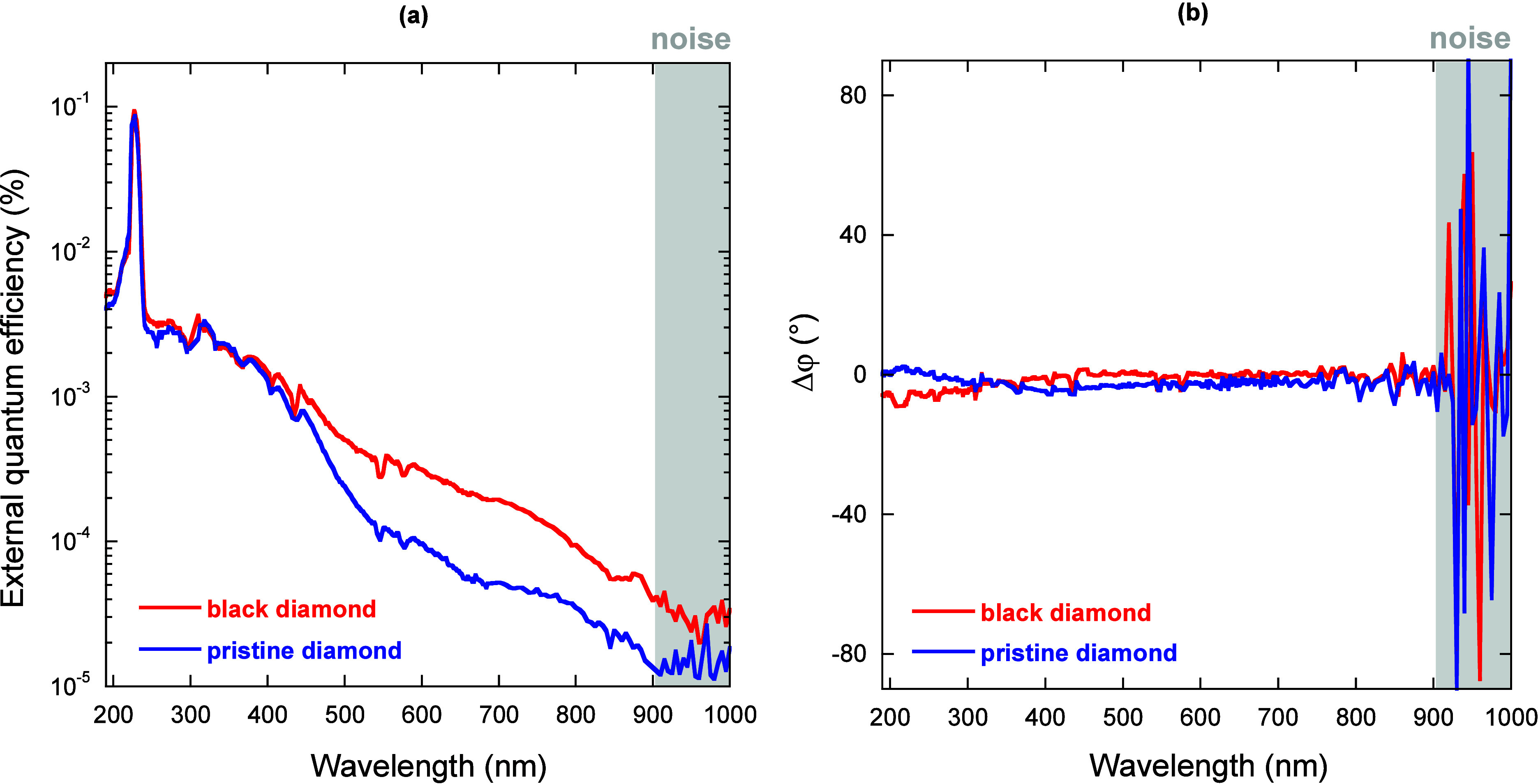
External quantum efficiency (a) and photocurrent signal phase shift (b) of the black diamond sample (red curves) and the pristine sample (blue curves) measured as a function of the monochromator output wavelength. Gray boxes indicate the wavelength range in which the measured signal amplitudes are below the noise level.

For λ < 400 nm no enhancement of EQE is observed, whereas for λ > 900 nm, both signals are completely unlocked (i.e., the lock-in amplifier does not sense the signal phase, as can be seen from Figure 3b for λ > 900 nm), because the measured photocurrents are below the background noise level. Moreover, it is worth noting that the phase shift Δφ, defined as the difference between the instantaneous phase and the reference phase recorded when the signal was locked for the first time, is constantly around 0°, implying no photon-induced building of space charge regions (Figure 3b).

To better highlight the photocurrent enhancement induced by defect engineering, we define the photoelectronic gain *k*_pe_ as the ratio between the photocurrent measured for black diamond to the one measured for the pristine sample. In addition, we define the ″electrically active” intermediate band (EIB) as a sub-bandgap energy interval in which *k*_pe_ > 1. The presence of an EIB is a direct demonstration of the effectiveness of the laser-induced defect engineering because it implies that the density of electrically active defects of the defect-engineered sample is higher than that of the nonengineered one. The photoelectronic gain is reported in Figure 4. We can observe that a primary EIB emerges, peaking at λ = 678 nm, where *k*_pe_ reaches its maximum value (*k*_pe_ ≈ 4). It is also worth noting a secondary EIB, peaking at λ = 248 nm, where however a significantly lower gain (*k*_pe_ ≤ 1.3) is achieved; in addition, with the solar irradiance being negligible at 248 nm, the secondary EIB is not functional to black diamond laser-induced defect engineering for solar cells. Conversely, the peak wavelength of the primary EIB corresponds to a photon energy of 1.83 eV (Figure 4b), thus perfectly within the range of maximum solar irradiance at Earth’s surface. Obviously, the photocurrent enhancement in the solar irradiance spectrum is still not high enough to state that black diamond is competitive to other materials used today in photovoltaics, since the conversion efficiency extrapolated for a black diamond photovoltaic cell is currently only 0.01% (Supporting Information, Supporting Note 1). Anyway, the optimization of black diamond in terms of density of electrically active defects and charge transport properties, combined with its application as a photon and heat converter, as in photon-enhanced thermionic emission devices,^23^ is highly promising.

**Figure 4:**
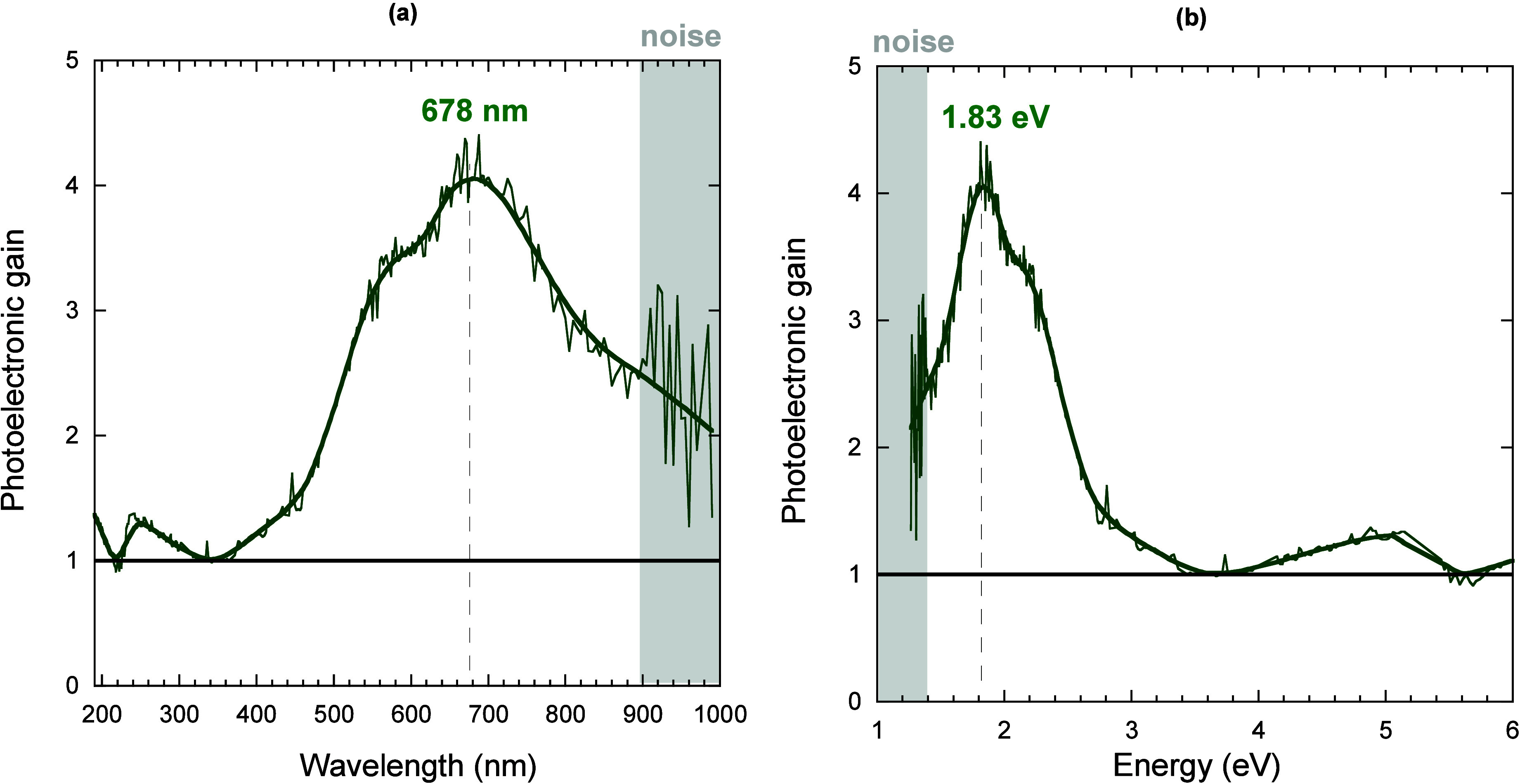
Photoelectronic gain calculated as a function of the monochromator output wavelength (a) and photon energy (b). Gray boxes indicate the wavelength and energy ranges in which the measured signal amplitudes are below the noise level. Thick solid lines are smoothing fitting curves of the experimental data, used to better highlight the peak wavelength and energy (indicated by the dashed vertical lines). Horizontal solid black lines indicate that *k*_pe_ = 1.

Once the presence of a primary EIB is highlighted, it is, however, not possible with room-temperature one-photon absorption techniques to deduce if it is located at 1.83 eV below the conduction band (CB) edge or above the valence band (VB) edge. Indeed, the absorbed photon can promote an electron either from the VB to the EIB (enabling hole conduction) or from the EIB to the CB (enabling electron conduction). In the latter case, it is necessary that the EIB is at least partially filled with electrons, and this can be obtained either through thermal excitation (which cannot be neglected at room-temperature) or through the absorption of a second photon. Therefore, to infer the position of the EIB within the bandgap by spectral photocurrent measurements, two techniques can be effectively used: (i) one-photon photocurrent measurements at extremely low temperatures^13^ (e.g., 4.2 K), aimed at ruling out electron conduction through thermally filled traps; (ii) two-photon sub-bandgap photocurrent measurements.

In this work, two-photon sub-band gap photocurrent measurements were performed, as will be described in the following section.

### Two-Photon Sub-Bandgap Photocurrent Measurements

The experimental setup for two-photon sub-band gap photocurrent measurements, shown in Figure 5, was obtained by modifying the setup arranged for one-photon experiments (Figure 2).

**Figure 5:**
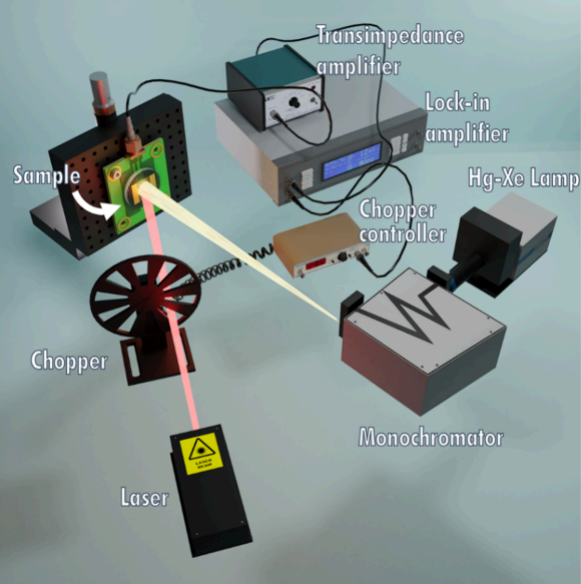
Sketch of the TPPC experimental setup with chopped laser light and continuous monochromator output light.

The sample is simultaneously irradiated by (i) the light produced by the Hg–Xe lamp, and subsequently monochromatized, spanning a wavelength range 190–1000 nm (photon energy *E*_mono_ = 1.24–6.52 eV); (ii) a laser beam with a fixed sub-bandgap wavelength. Specifically, two types of continuous-wave lasers have been used: a red laser, emitting at 635 nm (photon energy *E*_laser_ = 1.95 eV), and an IR laser, emitting at 816 nm (photon energy *E*_laser_ = 1.52 eV). As can be seen, only the laser light is modulated by the mechanical chopper, whereas the monochromator (MC) light irradiates the sample continuously. Therefore, only the component of the photocurrent signal induced by the laser beam is directly measured by the lock-in amplifier: this implies that, being *E*_laser_ lower than the diamond energy bandgap *E*_gap_ = 5.46 eV, photocurrent response can only be triggered if a sub-bandgap trap state is present at an energy distance *E*_trap_ ≤ *E*_laser_ from the conduction (or valence) band edge. The continuous MC light does not directly produce a signal but only serves as a pump for electrons, promoting them from the sub-band gap trap state to the CB or from the VB to the sub-band gap trap state (in the case of empty traps). In both cases, the simultaneous absorption of a laser photon triggers a two-photon sub-band gap photocurrent response by activating the first (trap filling) or the second (trap emptying) transition, respectively.

Figure 6 reports the results of TPPC experiments performed with the red laser beam on the pristine sample (blue curve) and on the black diamond sample (red curve). As can be seen, a constant current (hereinafter referred to as the “baseline” photocurrent) is recorded in the 500–1000 nm wavelength range for both the samples (Figure 6a); it is a perfectly “locked” signal, i.e., a constant phase shift is measured by the lock-in preamplifier (Figure 6b), and it is about 1 order of magnitude higher than the background noise (Supporting Information, Supporting Note 2). Because the signal does not depend on the wavelength of the monochromator output photon, and after having excluded other spurious signal sources when evaluating the background noise, we can infer that it is due to the photocurrent directly generated by the laser beam by one-photon absorption. We recall here that one-photon sub-bandgap photocurrent can occur in both the black and the pristine diamond because they both have a certain density of electrically active defects, corresponding to an energy distance *E*_t_ < *E*_laser_ = 1.95 eV from the extended band edges, that can be usefully exploited by the red laser to generate photocurrent through one-photon absorption. Conversely, two-photon sub-bandgap photocurrent response must be excluded for λ > 500 nm because *E*_mono_+ *E*_laser_< *E*_g_.

**Figure 6:**
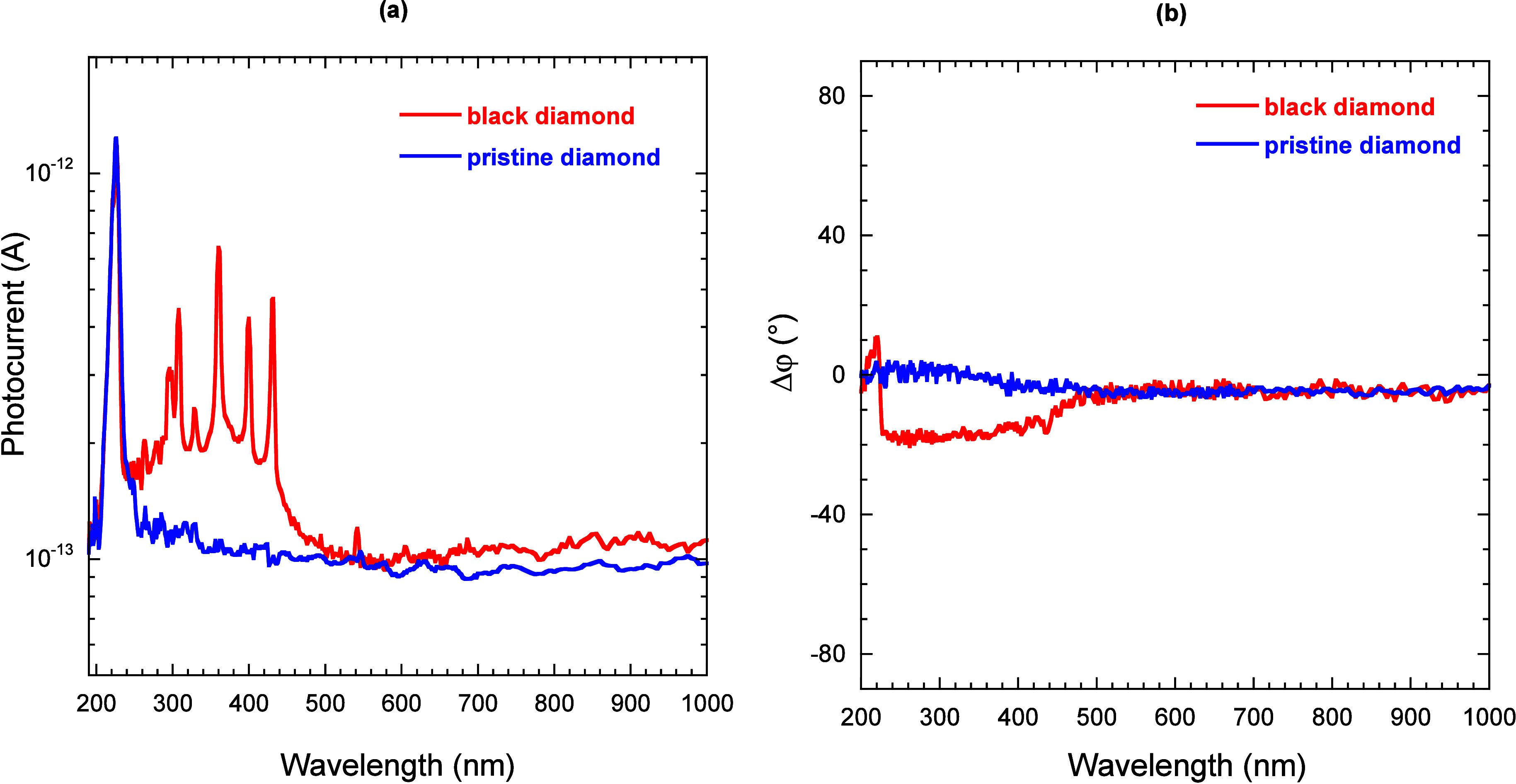
Photocurrent amplitude (a) and phase shift (b) measured for the black diamond sample (red curves) and the pristine diamond sample (blue curves) as a function of the monochromator output wavelength in the case of TPPC experiments with a red laser (635 nm).

By inspecting Figure 6a in the 500–1000 nm range, we are also able to infer the location of the EIB within the bandgap. As mentioned previously, there are two possible hypotheses:

1. If the EIB is 1.83 eV above the VB edge, the laser can generate a hole current by promoting electrons to EIB directly from the VB. Aimed at comparing the black diamond to the pristine one, let us refer to the single energy level at 1.83 eV, which in the case of black diamond is the centroid of the EIB. At a laser power of 5 mW, the rate of emitted photons is about 1.6 × 10^16^ s^–1^, of which the black diamond absorbs more than 90%, whereas the pristine one absorbs about 30%.^4^ According to the Fermi–Dirac distribution, before irradiation, the level is partially occupied by electrons thermally excited from the VB (the occupation probability is however unknown, because it is necessary to exactly locate the Fermi level). However, regardless of the initial occupancy state of the energy level in both the black and the pristine diamond, the density of absorbed photons is high enough to completely fill the level with electrons, leading to full occupation. Therefore, the photocurrent measured for the black diamond is higher than that measured for the pristine one, being higher the density of electrons (and consequently of holes) corresponding to level saturation.
2. If the EIB is 1.83 eV below the CB edge, the laser can generate an electron current by drawing electrons from the EIB and promoting them to the CB. However, level saturation is not possible in this case because the laser can only exploit the electrons thermally excited from the VB, the density of which is however very low at room temperature if we consider the high energy distance between the EIB and the VB (about 3.63 eV). As a result, the hole current produced by filling with electrons all the other empty states available to the laser photons (i.e., lying within 1.95 eV from the VB) overshadows the small electron current generated by exploiting the EIB. Therefore, the photocurrents measured for the black diamond and the pristine one are similar, with the photocurrent gain always close to unity outside the EIB (Figure 4b).

On the basis of the previous considerations, by observing the measured photocurrents for λ > 500 nm, we can conclude that the EIB is most likely located at 1.83 eV below the CB edge. Conversely, when λ < 500 nm, the photocurrent measured for the black diamond is higher than the one recorded from the pristine one, which is substantially constant and equal to that measured for λ > 500 nm: this strengthens the hypothesis on the location of the EIB below the CB and confirms the presence of a laser-induced one-photon photocurrent in the pristine diamond. More interestingly, in the case of black diamond, the photocurrent is clearly modulated by the intensity of the monochromator output (the peaks in the 300–400 nm range are indeed related to the emission lines of the Hg–Xe lamp). This can only be explained by the presence of TPPC in the black diamond sample: more specifically, the monochromator output photons trigger the first electronic transition from the VB to the EIB, whereas the red laser promotes electrons from the EIB to the CB. The increase of photocurrent starts indeed only when the monochromator photon energy is high enough to provide the EIB with a significant density of electrons, which are then released and promoted to the CB by the laser photons, producing a signal that adds to the “baseline” photocurrent. This is the reason why, despite the huge difference between the laser power (5 mW) and the lamp power (few tens of μW maximum), the signal is modulated by the monochromator output, which is the limiting factor of the TPPC signal: indeed, the TPPC signal clearly emerges from the “baseline” photocurrent only if the EIB is populated enough by the electrons pumped by the monochromator light. In addition, as can be seen from Figure 6b, the signal phase shift slightly decreases down to about −20° in the case of black diamond when λ < 500 nm; this is consistent with a partial electron filling of the EIB triggered by monochromator light absorption, inducing a negative charge accumulation. To our knowledge, this is the first direct experimental demonstration of TPPC in bulk materials with deep-level defects conceived for intermediate-band solar cells.

It is finally worth observing from Figure 6a the peak recorded at λ = 227 nm for both the black and the pristine diamond samples, which is due to a strong enhancement of the laser beam photocurrent generation by one-photon absorption. Indeed, when the monochromator wavelength approaches 227 nm (corresponding to the diamond bandgap), a huge number of interband transitions occur, which however cannot be detected by the lock-in amplifier because the monochromator light is not modulated. Nevertheless, a strong nonequilibrium condition is established in which the Fermi level *E*_F_ splits into two quasi-Fermi levels *E*_F*n*_ and *E*_F*p*_ for electrons and holes, respectively. Let us refer to electrons (but the discussion can be easily extended to holes). According to the Fermi–Dirac distribution, the probability for a trap with energy *E*_t_ to be filled with an electron is given by *f*_*t*_ = 1/(1 + exp­((*E*_t_ – *E*)/*kT*)), where *k* is the Boltzmann’s constant, *T* is the temperature, and *E = E*_F_ or *E* = *E*_F*n*_ under equilibrium or nonequilibrium conditions, respectively. Being always *E*_F*n*_ > *E*_F_, the trap occupancy probability is therefore higher when nonequilibrium conditions are triggered, in the specific case of photogeneration-induced nonequilibrium, there is a higher density of free electrons getting trapped during transport toward the collecting electrode, especially into shallow traps very close to the CB edge. However, the occupied shallow trap levels can be easily depopulated by the red laser, and trapped electrons can be released to the CB. As a consequence, the signal produced by the laser by one-photon absorption is strongly enhanced when interband transitions are triggered, because the laser has a much larger reservoir of occupied sub-band gap states available for photogeneration.

By a more detailed inspection of the TPPC photocurrent amplitude as a function of the monochromator output energy (Figure 7), it can be clearly seen that the black diamond photocurrent peaks at *E*_mono_ = 3.49 eV, i.e., at an energy distance of 1.97 eV from the bandgap, which is compatible with the photon energy of the red laser (*E*_laser_ = 1.95 eV). This can be understood by considering that the EIB, even if peaking at *E*_EIB_ = 1.83 eV, is quite broad (about 1.5 eV, Figure 4b); therefore, it is reasonable that the TPPC photocurrent peak slightly upshifts (or downshifts) with respect to *E*_EIB_, depending on the laser energy *E*_laser_.

**Figure 7:**
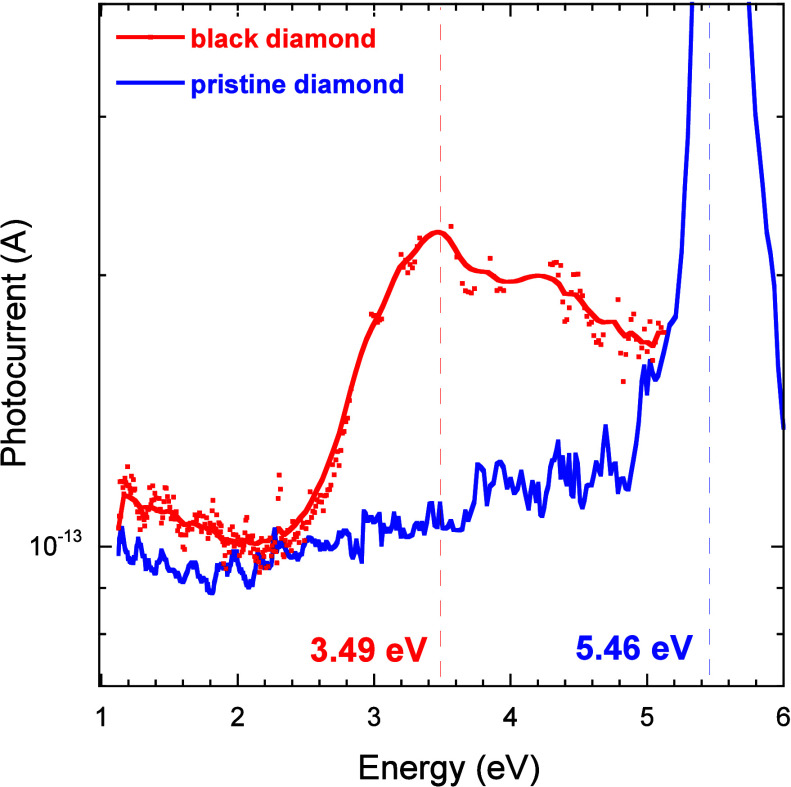
Photocurrent amplitude measured for the black diamond sample (red curve) and the pristine diamond sample (blue curve) as a function of the monochromator output photon energy in the case of TPPC experiments with a red laser (635 nm). Red and blue dashed lines indicate the positions of the photocurrent maximum values. In the case of black diamonds, red dots are related to experimental data, whereas the red solid line is a smoothing fitting curve used to exclude the secondary peaks corresponding to the Hg–Xe lamp emission lines from the estimation of the photocurrent peak position.

To confirm this hypothesis, we performed a second set of TPPC experiments using an IR laser (wavelength 816 nm, photon energy 1.52 eV). Results are reported in Figure 8. As can be inferred from the photocurrent amplitude spectrum (Figure 8a), two-photon sub-bandgap photocurrent is also measured under IR laser irradiation, but the signal amplitude in the TPPC regime (λ < 500 nm) is lower than the case of the red laser. This can be explained by considering that the IR laser photon energy (1.52 eV) is lower than the peak energy of the EIB (*E*_EIB_ = 1.83 eV), whereas the red laser energy (1.95 eV) is higher than *E*_EIB_: as a consequence, the IR laser has a lower availability of trapped electrons to trigger the second transition (from the EIB to the CB) and enable TPPC. This is also confirmed by a slightly lower value of the signal phase shift (Figure 8b) in the TPPC range (about −15°), indicating a smaller accumulation of negative charge.

**Figure 8:**
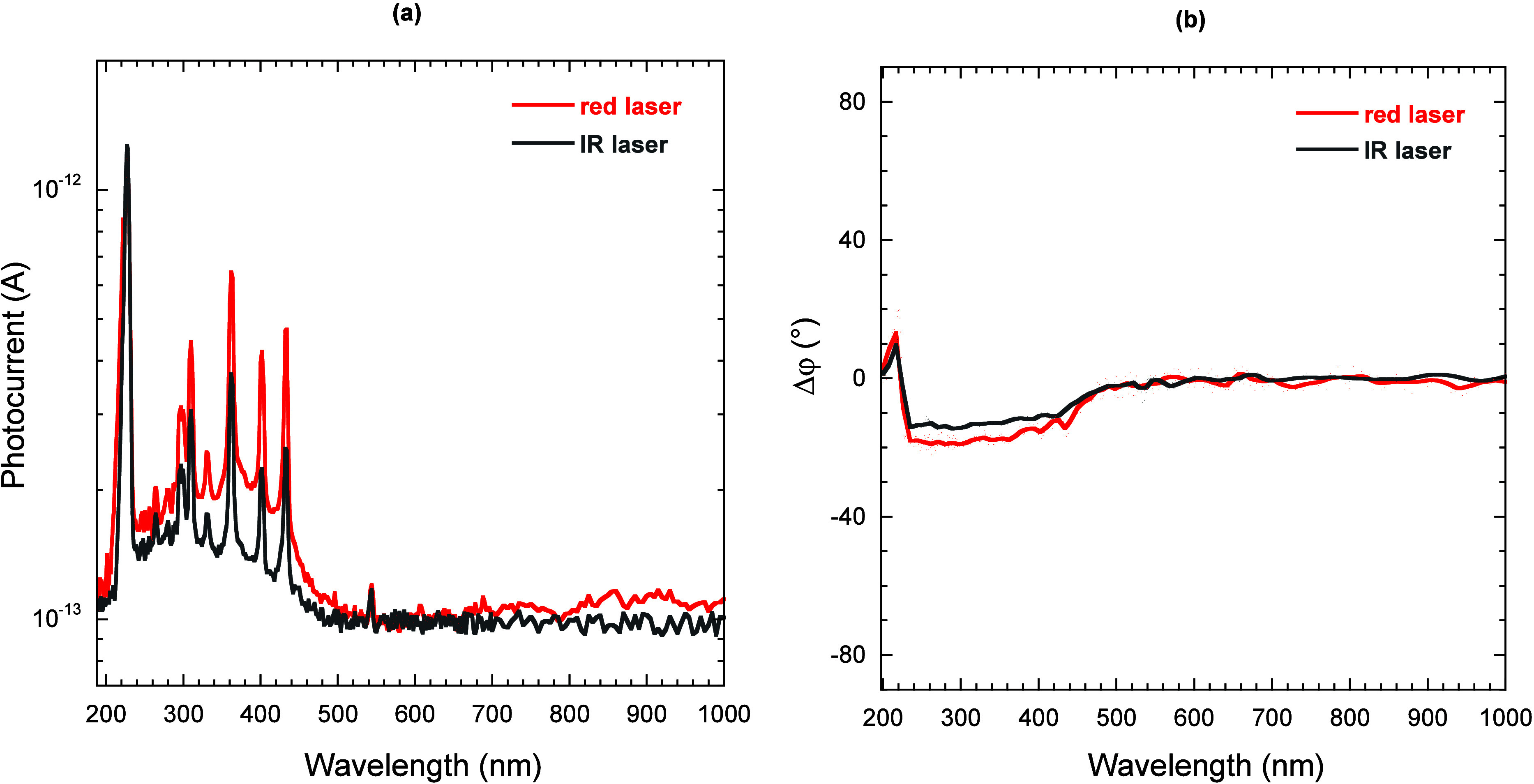
Photocurrent amplitude (a) and phase shift (b) measured for the black diamond sample as a function of the monochromator output wavelength in the case of TPPC experiments with a red laser (635 nm, red curves) and an IR laser (816 nm, gray curves).

Figure 9 clearly shows that the sub-bandgap photocurrent measured in the case of IR laser irradiation peaks at a lower monochromator output wavelength (i.e., at a higher energy) than the case of the red laser because the monochromator output photons, responsible for the first transition (from the VB to the EIB), must compensate for the lower laser energy. As in the case of the red laser, the sub-bandgap photocurrent peak energy (*E*_mono_ = 3.97 eV) is compatible with the energy difference between the bandgap energy and the IR laser energy (*E*_g_ – *E*_laser_ = 5.46–1.52 = 3.94 eV). In addition, it is worth noting that, as expected, the EIB peak lies between the two sub-bandgap photocurrent peaks recorded under red and IR laser irradiation, respectively.

**Figure 9:**
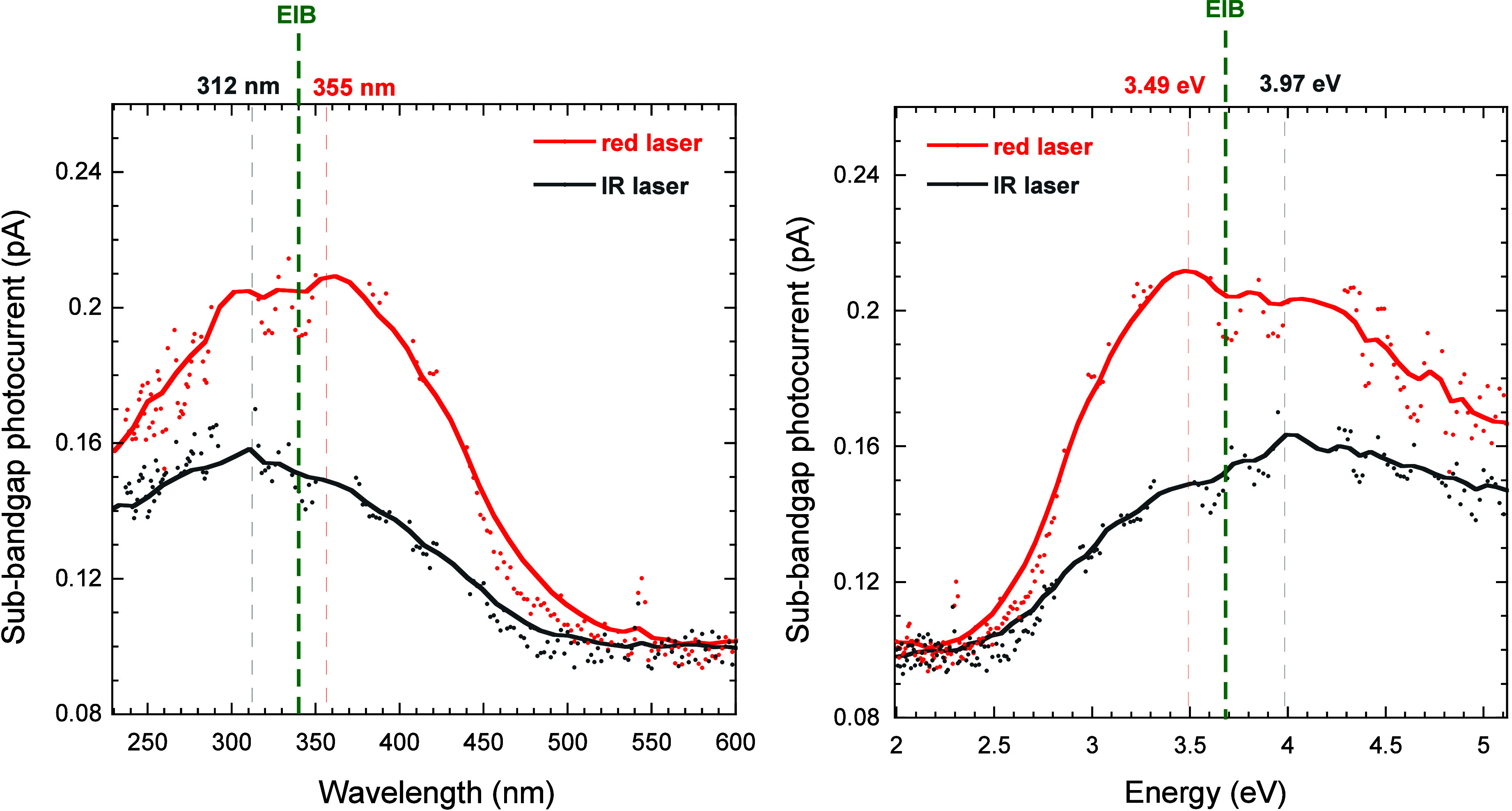
Sub-bandgap photocurrent amplitude measured for the black diamond sample as a function of the monochromator output wavelength (left) and energy (right) in the case of TPPC experiments with a red laser (635 nm, red curves) and an IR laser (816 nm, gray curves). Red and gray dashed lines indicate the position of the photocurrent maximum values, whereas the green dashed line indicates the position of the EIB peak. Dots are related to experimental data, whereas solid lines are smoothing fitting curves, used to exclude the secondary peaks corresponding to the Hg–Xe lamp emission lines from the estimation of the photocurrent peak positions.

In another experiment, aimed at providing a further demonstration of TPPC in surface-nanotextured black diamond, we simultaneously irradiated the sample with the monochromatized Xe–Hg lamp and the red laser, but the mechanical chopper was positioned in front of the monochromator output slit (Figure 10a); therefore, only the component of the photocurrent signal induced by the monochromator was directly measured by the lock-in amplifier. Normalized EQE results, directly derived from the modulated photocurrent measured under different laser power values (ranging from 0 to 5 mW), are reported in Figure 10b.

**Figure 10:**
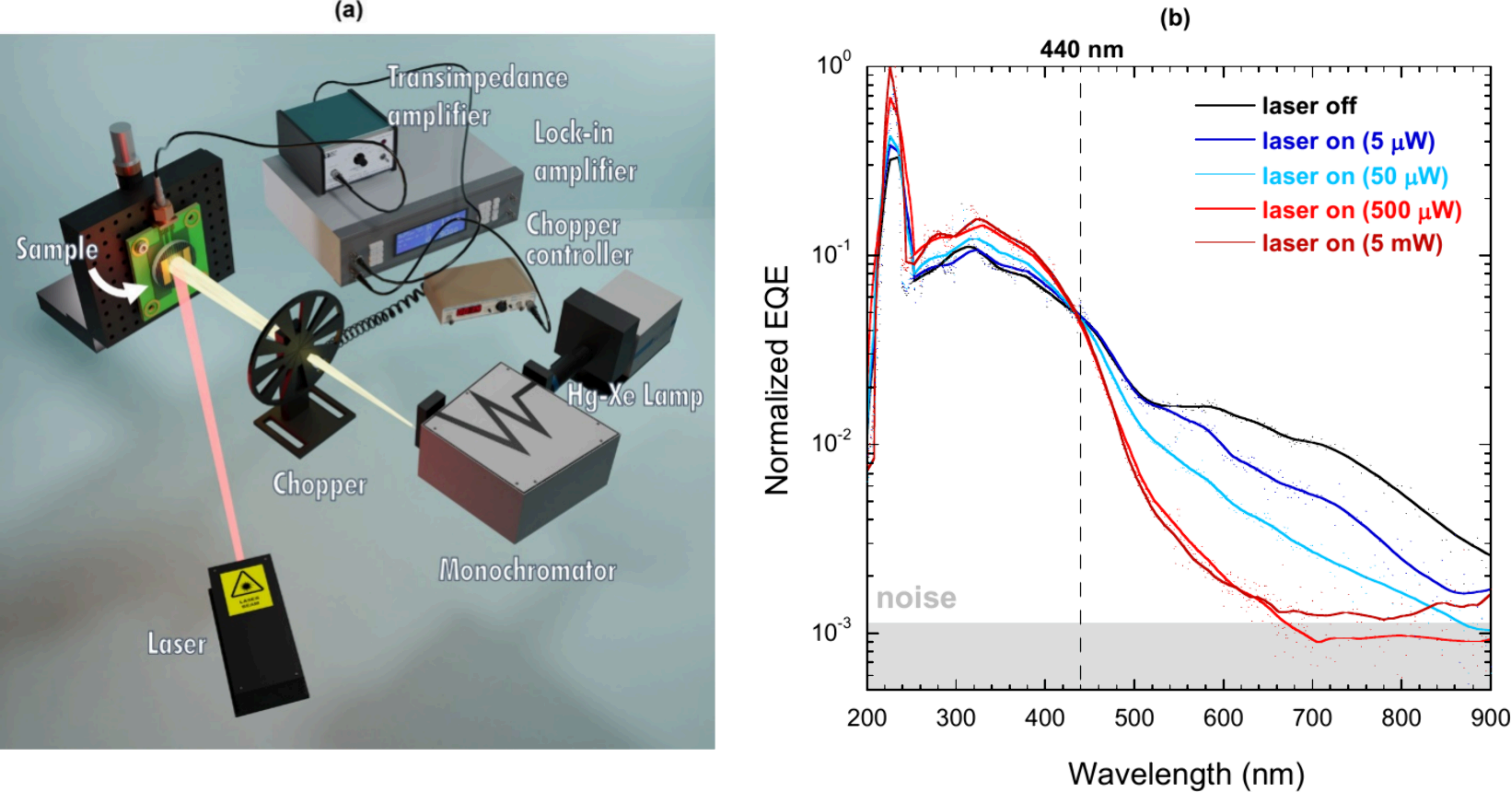
(a) Sketch of the TPPC experimental setup with chopped monochromator output light and continuous laser light. (b) Normalized external quantum efficiency of the black diamond sample as a function of the monochromator output wavelength at different laser power values (red laser, 635 nm wavelength, 1.95 eV energy). The gray box indicates the background noise level.

As can be seen, the effect of laser irradiation depends on the monochromator output wavelength:

1. When λ > 440 nm (*E*_mono_ < 2.82 eV), EQE strongly decreases with increasing laser power. In this case, TPPC must be excluded, being always *E*_mono_ + *E*_laser_ < *E*_gap_. One-photon photocurrent response is however possible due to the small amount of sub-bandgap defect states thermally populated by electrons (in accordance with the Fermi–Dirac distribution at room temperature), which can be directly promoted to the CB by the monochromator output photons. The concurrent absorption of laser output photons depopulates such states, emptying the reservoir of electrons available to the monochromatized light and quenching the photocurrent; this is obviously more noticeable at a higher laser power. It is worth stressing here that, if the defect-related energy is lower than 1.95 eV, the laser-induced depopulation of the defect states may promote electrons into the CB, which however cannot be detected by the lock-in amplifier, with the laser light not modulated by the optical chopper.
2. When λ < 440 nm (*E*_mono_ > 2.82 eV), EQE slightly increases with increasing laser power. In this case, TPPC is possible by exploiting defect states lying at energy distance *E*_laser_ = 1.95 eV from the VB. Specifically, absorbed laser output photons pump electrons from the VB to the defect states, then the monochromator output photons induce the second transition to the CB, thus triggering the TPPC signal. This is consistent with the presence of the secondary and less intense EIB in the 3.70–5.46 eV range, peaking at λ = 248 nm (Figure 4a). Obviously, in this case a higher laser power implies a higher density of electrons available for photoconduction, resulting in a higher photocurrent.

The different extent of EQE variation (strong decrease when λ > 440 nm, slight increase when λ < 440 nm) can be understood by considering the peak position of the primary EIB at 678 nm (Figure 4a), reflecting into a higher density of electrically active defects in the 400–900 nm range available for the absorption of laser photons and the subsequent quenching of photoconduction. Conversely, the low increase of EQE for λ < 440 nm is obviously related to the low photoelectronic gain introduced by the secondary EIB. In both cases, however, saturation of the available defect states can be observed when laser power is slightly above 500 μW.

## Conclusions

Two-photon sub-bandgap photocurrent (TPPC) response was demonstrated for the first time in a bulk semiconductor with deep-level defects. Specifically, an electrically active intermediate band (EIB) was experimentally observed in a double-nanotextured black diamond film obtained from native semitransparent polycrystalline CVD diamond by surface femtosecond laser treatment and conceived to be used as a photovoltaic solar absorber. This work, by experimentally confirming that TPPC is the actual physical mechanism of sub-bandgap photocurrent generation in solar energy conversion devices based on black diamond technology, provides the key to a complete understanding of their operating principle, which had only been speculated before. In addition, it is worth stressing here that the obtained results are of general validity and that the employed technique of investigation can be profitably applied to other defect-engineered semiconductors.

Results also indicate that the EIB is centered at an energy distance of 1.83 eV from the conduction band edge; however, to confirm this hypothesis, further investigations are needed, exploiting, for instance, unipolar techniques able to disentangle the different contributions of holes and electrons to photocurrent. In addition, taking the cue from these preliminary but significant results, further research activities are necessary to find possible correlations between the position of the EIB within the diamond energy bandgap and the laser processing parameters (e.g., wavelength, accumulated fluence), as well as with the type of diamond substrate (e.g., single-crystals, large- or small-grained polycrystals with different structural qualities), with the aim of upgrading the readiness level of defect-engineered black diamond technology for solar energy conversion.

## Supplementary Material



## Notes

Calculation of the power conversion efficiency and evaluation of the background noise (PDF)
