# Supplementary material for: Two-Photon Sub-Bandgap Photocurrent in Surface-Nanotextured Black Diamond Films for Solar Energy Conversion
Source: ACS Photonics. 2025 Aug 11;12(11):5874–83. doi: 10.1021/acsphotonics.5c00722 (PMC12637860; doi:10.1021/acsphotonics.5c00722)
Supplement: Supplementary file 1 [file ph5c00722_si_001.pdf]

Supporting Information

Two-Photon Sub-Bandgap Photocurrent in Surface-Nanotextured Black Diamond Films for Solar Energy Conversion

M. Girolami[*,a], A. Bellucci[a], M. Mastellone[b], S. Orlando[b], S. Pettinato[a,c], V. Serpente[a], S. Salvatori[a,c], D. M. Trucchi[a]

[a]*DiaTHEMA Lab, Istituto di Struttura della Materia, Consiglio Nazionale delle Ricerche (ISM - CNR), Sede Secondaria di Montelibretti, Strada Provinciale 35D 9, 00010 Montelibretti, Roma, Italy*

[b]*Istituto di Struttura della Materia, Consiglio Nazionale delle Ricerche (ISM - CNR), Sede Secondaria di Tito Scalo, Area Industriale - Contrada S. Loia, 85050 Tito Scalo, Potenza, Italy*

[c]*Faculty of Engineering, Università degli Studi Niccolò Cusano, Via don Carlo Gnocchi 3, 00166 Roma, Italy*

[*]Corresponding author: Marco Girolami (Tel: +39 06 90 672 237, e-mail: marco.girolami@ism.cnr.it)

Supporting Note 1: Calculation of the Power Conversion Efficiency

Let us consider a hypothetical standard photovoltaic cell formed by the non-optimized black diamond sample used for this work. Since 1 sun irradiation corresponds to a power density of 0.1 W cm^{-2}, the concentration ratio of our monochromator output light (in the range of 1 μW cm^{-2}) is equivalent to about 10^{-5} suns. Under this excitation intensity, we derived the spectral responsivity $S(\lambda)$ of the device:

$$S(\lambda) = EQE(\lambda) \cdot \frac{q\lambda}{hc}$$

where $EQE(\lambda)$ is the external quantum efficiency reported in Figure 3a (red curve) as a function of the excitation wavelength λ, q is the electron charge, h is the Planck's constant and c is the speed of light in vacuum.

Then we obtained the hypothetical photocurrent per unit wavelength $I_{ph}(\lambda)$ produced under 1 sun irradiation:

$$I_{ph}(\lambda) = S(\lambda) \cdot W_{AM1.5}(\lambda)$$

where $W_{AM1.5}(\lambda)$ is the standard solar spectral irradiance[1].

By supposing a $V_{op} = 2.5$ V open-circuit voltage, which is a value compatible with the wide energy bandgap of diamond (5.46 eV), we calculated the approximate output power $W_{out}(\lambda)$ per unit wavelength as:

$$W_{out}(\lambda) = V_{op} \cdot I_{ph}(\lambda)$$

The output power per unit wavelength is reported in Figure S1.

Finally, the power conversion efficiency of the cell was obtained by the ratio of the integral area of the $W_{out}(\lambda)$ curve to that of the standard solar spectral irradiance $W_{AM1.5}(\lambda)$ curve:

$$\eta(\%) = 100 \cdot \frac{\int_0^\infty W_{out}(\lambda)d\lambda}{\int_0^\infty W_{AM1.5}(\lambda)d\lambda} = 0.01\%$$

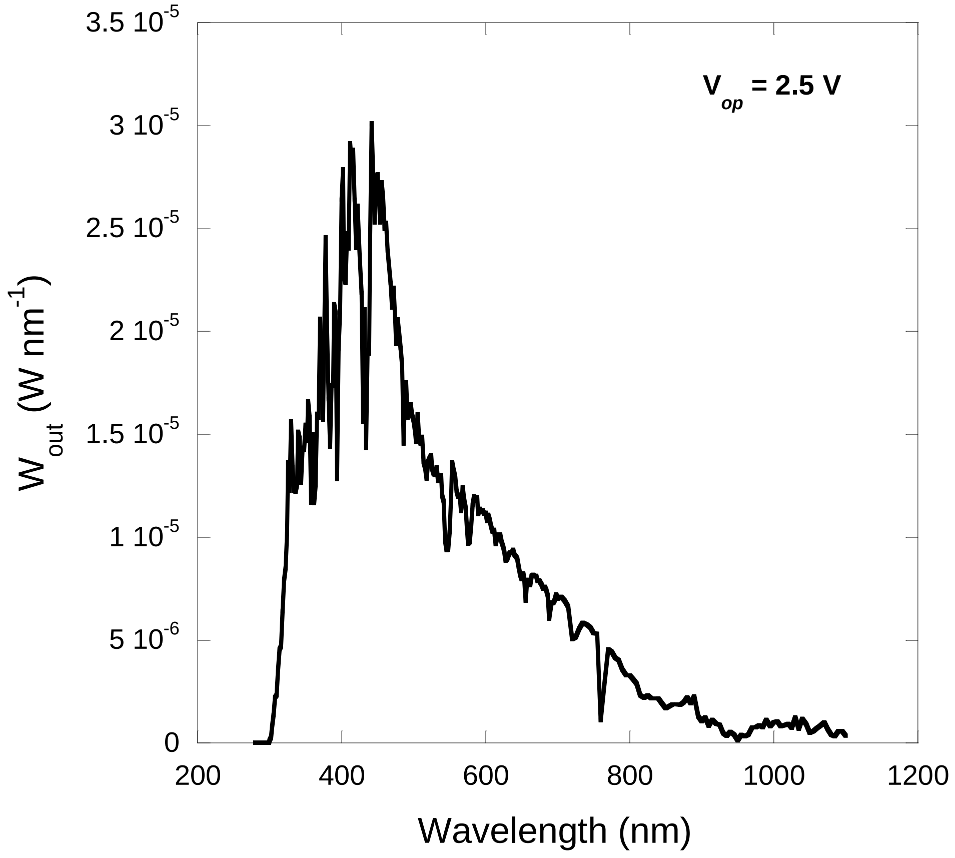

Figure S1. Output power per unit wavelength produced by a hypothetical photovoltaic cell based on a non-optimized black diamond sample under 1 sun irradiation.

Supporting Note 2: Evaluation of the Background Noise

To evaluate the background noise before TPPC experiments (Figure 5), current measurements were performed on both the samples with the laser off (but the chopper on) as the monochromator illuminated the samples with an unmodulated stream of photons of different energies[2]. In the absence of laser-induced transitions, this should not produce any signal. In addition, possible spurious signals, produced for instance by a portion of monochromatized light reflected from the spinning chopper blades, should be detected. Experimental results are reported in Figure S2.

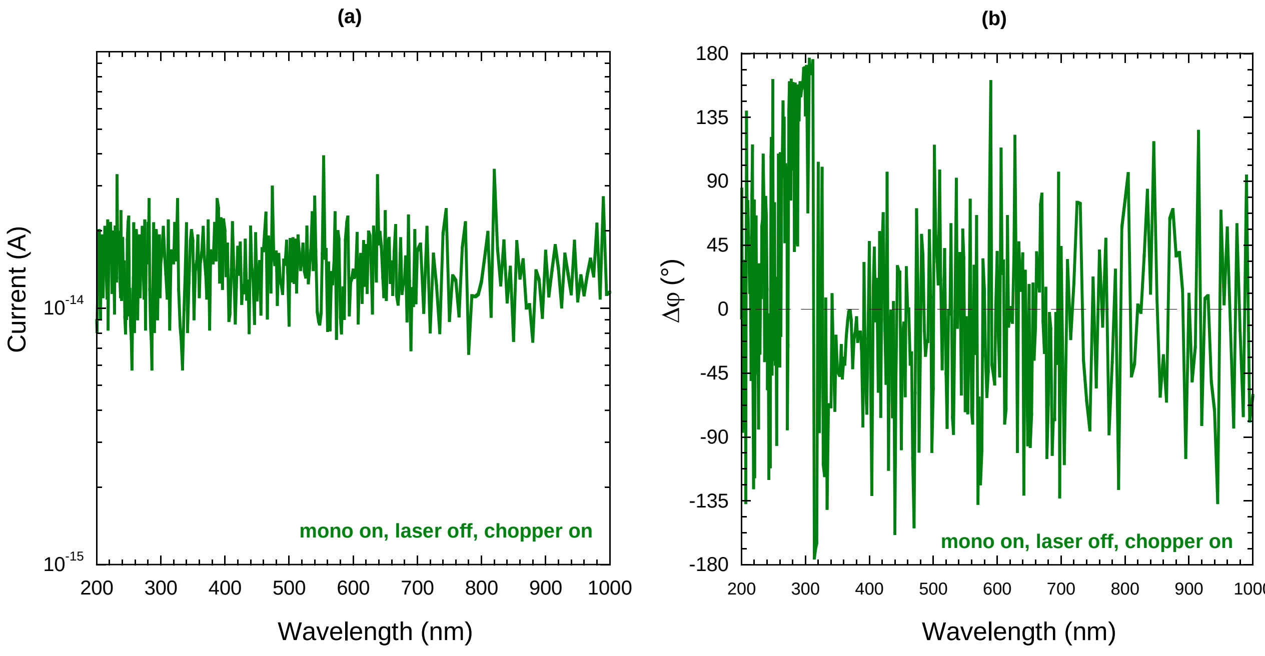

Figure S2. Current amplitude (a) and phase shift (b) measured for the black diamond sample as a function of the monochromator output wavelength for the background noise evaluation. Similar results have been obtained for the pristine sample.

As can be seen from Figure S2a, the average measured current is slightly above 10^{-14} A (equal to the sensitivity of the lock-in amplifier), which is about one order of magnitude lower than the baseline photocurrent (Figure 6a) recorded during TPPC experiments. More significantly, the phase shift (Figure S2b) is not constant, but oscillates continuously in the full (-180°, +180°) range, indicating that the measured current is not "locked", and that there is no signal detected by the lock-in amplifier.

References

(1) Reference Solar Spectral Irradiance: ASTM G-173 ASTM, https://www.nrel.gov/grid/solar-resource/spectra-am1.5

(2) Martí, A.; Antolín, E.; Stanley, C.R.; Farmer, C.D.; López, N.; Díaz, P.; Cánovas, E.; Linares, P.G.; Luque, A. Production of Photocurrent due to Intermediate-to-Conduction-Band Transitions: A Demonstration of a Key Operating Principle of the Intermediate-Band Solar Cell, *Phys. Rev. Lett.* **2006**, *97*, 247701. https://doi.org/10.1103/PhysRevLett.97.247701
